# Audit Review of Psychotropic Medication of the Caerphilly Psychiatry Community Learning Disability Caseload

**DOI:** 10.1192/bjo.2026.11671

**Published:** 2026-06-30

**Authors:** Myo Tay Zar Lin, Abigail Swift, David Medhurst

**Affiliations:** NHS, Newport, United Kingdom

## Abstract

**Aims:**

This audit aimed to review patterns of psychotropic prescribing within a community LD psychiatry service in Caerphilly, focusing on medication type, dosage, documented clinical indication, and trends in medication change over time.

Psychotropic medications play a central role in the management of mental health conditions among individuals with Learning Disability. However, their use in this population requires careful consideration due to increased vulnerability to adverse effects, polypharmacy, and challenges in monitoring efficacy and safety. National guidelines, including those from NICE and the Royal College of Psychiatrists, emphasize the importance of regular review, clear documentation of indications, and adherence to best practice standards to minimize inappropriate prescribing.

**Methods:**

A retrospective review of clinic and home visit letters for all patients seen by the community LD psychiatry service during the audit period was undertaken. Data were collected by 3 doctors on level of LD, presence of autism spectrum disorder (ASD) or attention deficit hyperactivity disorder (ADHD), psychiatric diagnoses, psychotropic medications prescribed, antipsychotic dose expressed as a percentage of the British National Formulary (BNF) maximum, and medication changes over the audit period.

**Results:**

A total of 117 patients were included. 114 (97.4%) were prescribed at least one psychotropic medication. 77 patients (65.8%) were prescribed antipsychotics, mostcommonly risperidone (n=37). Antipsychotic doses ranged from 1.5% to 100% of the BNF maximum, with 47 patients (61.0%) prescribed doses at or below 25% of the maximum.

A clear mental health diagnosis supporting antipsychotic use was documented in 23 of 77 patients (29.9%), while behaviours that challenge were documented in a further 15 patients (19.5%). No clear indication for antipsychotic prescribing was recorded in the remaining 39 patients (50.6%). Antidepressants were prescribed to 44 patients; anxiety or depressive disorders were documented in 16 patients (36.4%), while 8 patients (18.2%) had no documented indication.

Over the audit period, psychotropic medication remained unchanged in 57 patients (48.7%), increased in 27 patients (23.1%), reduced in 23 patients (19.7%), and switched or cross-titrated in 6 patients (5.1%).

**Conclusion:**

This audit demonstrates high rates of psychotropic prescribing within a community LD caseload, with substantial gaps in documentation of clinical indication, particularly for antipsychotic and antidepressant use. Although some medication reduction occurred, increases were more common than reductions. Improved documentation, structured medication review processes, and multidisciplinary approaches are required to support safe prescribing and align practice with national guidance.

